# Jost Steinhäuser: Telemedizin und eHealth. Das Wichtigste für Ärztinnen und Ärzte aller Fachrichtungen

**DOI:** 10.3205/zma001637

**Published:** 2023-09-15

**Authors:** Christian Vajda

**Affiliations:** 1 Medizinische Universität Graz, Univ.-Klinik für Psychiatrie, Psychosomatik und Psychotherapie, Klinische Abteilung für Medizinische Psychologie, Psychosomatik und Psychotherapie, Graz, Austria

## Bibliographical details

Jost Steinhäuser

**Telemedizin und eHealth. Das Wichtigste für Ärztinnen und Ärzte aller Fachrichtungen**

Publisher: Urban & Fischer in Elsevier

Year of publication: 2021, 160 pages, price: € 37,00

ISBN: 978-3-437-23545-0

## Review

The digitalization of the healthcare system is progressing continuously. This changes the daily work processes as well as communicative and perceptual aspects in everyday healthcare work. This book, inspired by the years of the corona pandemic, takes on the different aspects of telemedicine and eHealth and attempts to give the reader a compact and practical point of view. Both the opportunities and the dangers of eHealth are discussed.

The authors dedicate to the evidence, the legal framework and a wide range of practice-relevant topics of digitalization. This includes, for example, communication, data security issues, the quality of apps or wearables, ethical and technical aspects and finally didactic ideas for training in health care.

The chapter structure is characterized by good clarity. In particular, the core statements give a short and concise overview at the beginning. Furthermore, where appropriate, tips and case studies are given and highlighted in colour. When the book was written, many of the authors were working on specific eHealth projects, a circumstance that is of benefit to the readers and can offer ideas for their own projects. Examples from neurological, internal medicine or even dermatological questions are specifically given.

A separate area is also dedicated to the possibilities regarding to practice management. This includes practice organization, strategic investment planning, electronic patient files and the use of social media.

Aside from the examples and theoretical background, the book also always addresses the opportunities and risks of digitalization. For example, the importance of the prevalence range for apps, possible effects on the future of the medical profession and the dangers of cyber attacks or ethical issues.

The readability is consistently good, although individual chapters are written more “technically” and could therefore demand a little more concentration from the reader.

Despite its only 160 pages, the book offers extensive ideas for interested people from all disciplines. However, as already noted in the title, the book can only be a first introduction to the subject and certain aspects such as communication or the consequences for the medical profession could be more elaborated. This also reflects the reality in which the discussion has so far been primarily determined by the technical aspects of telemedicine and eHealth as well as the possible administrative overload and relief. However, the interpersonal consequences (or how these will have to be taken into account at the training level in the future) still receive too little attention in the discourse. Beside this it is commendable that the authors take a look at it and do not ignore it.

## Competing interests

The author declares that he has no competing interests.

